# Outcomes in COVID-19 Patients with Acute Cholangitis: A Single-Center Retrospective Analysis

**DOI:** 10.3390/medicina60081354

**Published:** 2024-08-20

**Authors:** Deiana Vuletici, Bogdan Miutescu, Calin Burciu, Iulia Ratiu, Tudor Moga, Eyad Gadour, Alexandru Catalin Motofelea, Oana Koppandi, Roxana Sirli, Alina Popescu

**Affiliations:** 1 Department of Gastroenterology and Hepatology, “Victor Babes” University of Medicine and Pharmacy Timisoara, Eftimie Murgu Square 2, 300041 Timisoara, Romania; deiana.vuletici@umft.ro (D.V.); calin.burciu@umft.ro (C.B.); ratiu.iulia@umft.ro (I.R.); moga.tudor@umft.ro (T.M.); sirli.roxana@umft.ro (R.S.); popescu.alina@umft.ro (A.P.); 2 Advanced Regional Research Center in Gastroenterology and Hepatology, “Victor Babes” University of Medicine and Pharmacy Timisoara, 300041 Timisoara, Romania; 3 Department of Gastroenterology, Faculty of Medicine, Pharmacy and Dental Medicine, “Vasile Goldis” West University of Arad, 310414 Arad, Romania; oanakoppandi@yahoo.com; 4 Department of Gastroenterology, King Abdulaziz Hospital-National Guard Health Affairs, Al Ahsa 31982, Saudi Arabia; eyadgadour@doctors.org.uk; 5 Department of Medicine, Zamzam University College, Khartoum 11113, Sudan; 6 Department of Internal Medicine, Faculty of Medicine, “Victor Babeș” University of Medicine and Pharmacy Timisoara, Eftimie Murgu Square 2, 300041 Timisoara, Romania; alexandru.motofelea@umft.ro

**Keywords:** acute cholangitis, COVID-19, biliary drainage, microorganisms

## Abstract

*Background and Objectives*: This study aimed to assess the impact of coronavirus disease 2019 (COVID-19) on patients with acute cholangitis (AC) by comparing outcomes, complications, and hospital stays in a tertiary Gastroenterology department. *Materials and Methods*: This retrospective observational cohort study was conducted in a tertiary gastroenterology department, collecting data from all AC and AC + COVID-19 patients between April 2020 and February 2022. Data included clinical and demographic information, COVID-19-specific details, acute cholangitis presentation, medical records, laboratory results, and interventions. AC was diagnosed using Tokyo Guidelines 2018 (TG18) criteria, with all patients undergoing bile culture sampling. *Results*: The study included 241 patients, 30 in the COVID group and 211 in the non-COVID group. The COVID group’s mean age was significantly higher (74.3 vs. 67.3 years, *p* < 0.009). Abdominal pain was more common in the COVID group (90% vs. 70.6%, *p* < 0.025). Length of hospital stay was longer for COVID patients (13.5 vs. 7.9 days, *p* < 0.001). COVID patients had higher incidences of malignant causes of AC, with pancreatic cancer being the most common (30%). *Pseudomonas* spp. was significantly more prevalent in COVID patients (16.7% vs. 5.7%, *p* = 0.028). *Conclusions*: Our study results show that COVID-19 affected the duration of hospitalization for patients with AC. Furthermore, this study presents observations regarding the impact of COVID-19 on AC, revealing differences in microbial profiles.

## 1. Introduction

Acute cholangitis (AC) is a potentially fatal condition requiring prompt detection and treatment [1]. This clinical syndrome arises when bacterial infections invade the normally sterile biliary system, typically in the context of a bile duct obstruction caused by choledocholithiasis, although it can also occur in patients with neoplasms and strictures [2]. Management of acute cholangitis hinges on the severity of the condition, with biliary drainage and antibiotics being the primary treatment modalities [3].

The Tokyo Guidelines 2018 (TG18) provide criteria for diagnosing AC, which include systemic inflammation, cholestasis, and imaging evidence of bile-duct abnormalities [4]. These guidelines also offer recommendations for the appropriate use of antimicrobial agents [5].

The emergence of coronavirus disease 2019 (COVID-19), caused by the SARS-CoV-2 virus, has profoundly impacted medical practices worldwide, including gastroenterology [6]. COVID-19’s rapid global spread has led to varied clinical presentations, from asymptomatic cases to severe illness, complicating the management of pre-existing conditions like acute cholangitis [7,8]. The need for emergency endoscopic procedures, such as endoscopic retrograde cholangiopancreatography (ERCP), persisted during the pandemic, despite overall reductions in gastrointestinal endoscopy volumes [9].

Notably, the pandemic did not significantly decrease the diagnosis of pancreaticobiliary cancers, nor did it alter the approach to ERCP for malignant and benign conditions [10]. However, patients with chronic liver disease who contracted COVID-19 faced higher risks of severe complications and mortality [11].

The liver’s susceptibility to SARS-CoV-2 infection due to the high expression of ACE2 receptors in cholangiocytes further complicates the clinical picture [12].

While liver function abnormalities are common in COVID-19 patients, significant liver impairment is rare [13]. Severe cases may experience liver injury due to immune-mediated inflammation, including cytokine storms and hypoxia-associated pneumonia [14].

This study aims to evaluate the impact of COVID-19 on patients with AC, comparing outcomes, complications, and length of hospital stay within a tertiary gastroenterology department. Additionally, the primary objective of this study was to characterize the microbiological profiles of bile aspirates collected during ERCP in patients with acute cholangitis. By exploring these interactions, this study seeks to enhance the understanding and management of AC in the context of the recent pandemic.

## 2. Materials and Methods

### 2.1. Study Design and Participants

Ethical approval was obtained from the Internal Review Board of “Pius Brinzeu” Emergency County Hospital of Timisoara, Romania, and patient confidentiality and data security were strictly maintained. This study was designed as a retrospective observational cohort single-center study conducted in a tertiary gastroenterology department to investigate the intersection of COVID-19 and acute cholangitis. It aims to elucidate the clinical characteristics, therapeutic interventions, and outcomes in patients with both conditions. Data were collected from patients with acute cholangitis, with or without COVID-19, between April 2020 and February 2022. Clinical and demographic data were systematically and retrospectively collected, including COVID-19-specific information, cholangitis presentation, medical records, laboratory results, radiological findings, and medical interventions. Additionally, patient-reported outcomes and complications were documented.

The study aims to characterize the microbiological profiles of bile aspirates from patients undergoing ERCP. The PICO elements are P (Population)—patients diagnosed with AC and COVID-19; I (Intervention)—comparison of clinical outcomes and microbiological analysis of bile; C (Comparison)—outcomes in patients with AC with and without COVID-19; and O (Outcomes)—clinical outcomes (e.g., hospital stay, complications), and microbiological profiles.

### 2.2. Inclusion Criteria

Patient inclusion criteria included testing for COVID-19 diagnosis through RT-PCR on nasopharyngeal swabs and clinical and imaging evidence of AC based on the TG18. Additionally, participants were required to be above 18 years of age and willing to provide informed consent. Patients excluded from the study were those with inadequate medical records or incomplete clinical data, inability to provide informed consent due to medical or psychiatric conditions, age below 18 years, antibiotics treatment for other medical conditions at the time of acute cholangitis diagnosis, post-ERCP perforation, cholangitis secondary to ERCP, or percutaneous or surgical drainage.

### 2.3. Diagnosis of AC

The TG18 criteria determined the AC diagnosis. Based on the TG18 criteria, diagnosing acute cholangitis (AC) relies on three essential factors: systemic inflammation, cholestasis, and imaging-detected bile-duct abnormalities. Systemic inflammation is a mandatory criterion typically identified by fever or elevated inflammatory markers, such as increased leukocyte count or elevated C-reactive protein levels. Despite significant advancements in diagnostic imaging techniques, direct imaging-based diagnosis of AC remains challenging, requiring a continued dependence on clinical and laboratory findings to confirm the disease [4]. Following admission, all patients were administered antibiotics according to the TG18 recommendations for the grade specified in the diagnosis of AC [5]. Culture media were used to identify microorganisms in bile samples.

Various diagnostic techniques were applied to address the cause of obstruction at admission. B-mode ultrasonography was initially performed, and when the diagnosis remained uncertain, further methods, such as endoscopic ultrasound (EUS), contrast-enhanced Computer Tomography (CE-CT), or magnetic resonance (CE-MRI), were used to assist in diagnosing and staging malignancies. Additional diagnostic confirmation was achieved by evaluating tumor markers and reviewing histopathological data derived from ERCP or EUS biopsies.

### 2.4. Therapeutic Approach

ERCP was performed using a therapeutic duodenoscope provided by Olympus Corp., Tokyo, Japan, to access the common bile duct through a guidewire. Under careful sedation management, a specialized anesthesia and intensive care team expertly administered a blend of midazolam, propofol, and fentanyl, following their internal protocols during ERCP procedures. The timing for ERCP was determined by evaluating the severity of the condition and adhering to the guidelines set forth by the endoscopists as outlined in the Tokyo Guidelines.

### 2.5. Data Acquisition and Study Variables

Meticulously documenting a broad range of variables enabled a thorough analysis throughout the study. The collected data encompassed various aspects, including patient demographics (gender, age), clinical observations (symptoms such as abdominal pain, jaundice, fever, chills), laboratory analysis, duration of hospitalization, severity according to the Tokyo guidelines, and microbial cultures of bile samples.

Additionally, we included COVID-19 patients with pneumonia, those who received antiviral treatment for COVID-19, as well as those with comorbidities such as cardiac pathology, type 2 diabetes mellitus, and chronic kidney disease in both groups of patients.

### 2.6. Statistic Analysis

Continuous variables were assessed for normality using the Shapiro-Wilk test. Normally distributed data were presented as means ± standard deviations (SD), while non-normally distributed data were summarized using medians with interquartile ranges (IQRs; 25th to 75th percentiles). Categorical variables were described as counts and percentages. Differences between groups for normally distributed continuous variables were assessed using Welch’s *t*-test for comparisons between two groups and one-way ANOVA for multiple groups, incorporating post-hoc tests (e.g., Tukey’s HSD) to pinpoint specific group differences. For non-parametric continuous data, the Mann-Whitney U and Kruskal-Wallis tests were applied for two and multiple groups, respectively, with subsequent Dunn’s post-hoc analyses as necessary. Categorical data comparisons were conducted using Chi-square tests or Fisher’s exact test when expected cell counts were below five. We used binomial logistic regression to identify independent predictors of study outcomes, carefully considering potential confounding factors. Before the main analysis, we checked for multicollinearity among the predictors to ensure the integrity of our regression model. The logistic regression model quantified the association between independent variables and the outcome variable through regression coefficients. These coefficients provided insight into the direction and importance of the effect of each predictor, with statistical significance determined by a *p*-value of less than 0.05. We applied a pseudo-R-squared measure alongside the Hosmer-Lemeshow goodness-of-fit check to evaluate our logistic regression model’s overall performance and suitability. Sample size calculations were conducted a priori to achieve a confidence level of 95% and a statistical power of 80% based on anticipated effect sizes and variance estimates derived from preliminary data. All statistical analyses were performed in R (version 3.6.3), leveraging the capabilities of several comprehensive packages within the Tidyverse for data manipulation and visualization, Finalfit for regression analyses, and other specialized packages (MCGV, Stringdist, Janitor, Hmisc) for various data processing needs.

## 3. Results

A total of 241 patients were included in this study. The etiology of AC is detailed in Table 1. Most patients in the benign group were diagnosed with choledochal lithiasis (43.6%). Malignant pathology was diagnosed in 53.3% of patients (*n* = 129/241), with pancreatic cancer (29%) being the most common cause. The mean age between the four groups showed statistical differences (*p* < 0.009). Patients in the COVID group had a mean age of 74.3 years (SD = 10.6), while patients in the non-COVID group had a mean age of 67.3 years (SD = 14.1). No gender differences were found between COVID and non-COVID patients (*p* = 0.539), as shown in Table 2. Abdominal pain was more common in the COVID group than in the non-COVID group (90% vs. 70.6%, *p* < 0.025). No differences were observed between the two groups in the incidence of fever (*p* = 0.246). Patients who tested positive for COVID-19 had an extended hospital stay of 13.5 days (SD = 6.6), compared to an average of 7.9 days (SD = 5.4) for patients not diagnosed with COVID-19.

A combination of correlation analysis, multiple regression analysis, and ANOVA was used to evaluate the relationships between hospitalization duration and factors such as sex, age, comorbidities (including cardiac pathology, Type 2 diabetes, and chronic kidney disease), pneumonia severity, and the probability of receiving COVID-19 treatment for patients with and without COVID-19.

The dataset included 30 COVID-19 patients, 15 of whom (50%) had pneumonia. Of those with pneumonia, 5 patients (16.7%) had minimal involvement, 7 patients (23.3%) had moderate involvement, and 3 patients (10%) had severe involvement. Additionally, 11 patients (36.7%) received COVID-19 treatment, while 19 (63.3%) did not.

Pearson correlation was used to explore the relationships between hospitalization duration and factors such as sex, age, and comorbidities in COVID-19 and non-COVID-19 groups. For the COVID-19 group, age (r = −0.131, *p* = 0.490) showed weak correlations with hospitalization duration. In the non-COVID-19 group, age (r = 0.155, *p* = 0.024) showed a weak but statistically significant positive correlation with hospitalization duration.

Multiple linear regression further quantified these relationships. In the COVID-19 group, Type 2 diabetes (β = 3.59, *p* = 0.142), gender (β = 1.38, *p* = 0.530), and age (β = −0.009, *p* = 0.937) were not statistically significant predictors of hospitalization duration. In the non-COVID-19 group, chronic kidney disease significantly affected hospitalization duration (β = 4.02, *p* = 0.063), and age also approached statistical significance (β = 0.053, *p* = 0.058). However, the model’s explanatory power was low, with an R-squared value of 0.039, indicating that these variables explained only 3.9% of the variance in hospitalization duration.

An ANOVA test was conducted to assess whether there were significant differences in hospitalization duration based on pneumonia severity for COVID-19 patients. The results indicated no statistically significant differences in average hospitalization duration across different levels of pneumonia severity (F = 0.192, *p* = 0.901). A binomial logistic regression was also performed to evaluate the association between hospitalization duration and the probability of receiving COVID-19 treatment. The regression analysis showed that hospitalization duration (coefficient = 0.0664, *p* = 0.265) was not significantly associated with the probability of receiving COVID-19 treatment.

The results presented in Table 3 show bile culture results associated with Tokyo severities and COVID-19 status, revealing different patterns of bacterial growth and culture sterility across various severity degrees. For patients with COVID-19, the highest proportion of sterile cultures was observed in mild Tokyo severity (60%), followed by severe cases (40%). No sterile cultures were identified in moderate-severity cases. In contrast, for patients without COVID-19 infection, sterile cultures were more evenly distributed, with a significant proportion observed in mild cases (40%), followed by moderate (33.3%) and severe grades (26.7%).

Grade I (mild) acute cholangitis had a prevalence of monomicrobial growth in patients with COVID-19 (25%) compared to those without COVID-19 (41.7%). For grade III (severe) acute cholangitis, a higher prevalence of COVID-19 was observed (50%) compared to non-COVID-19 cases (26.2%). Interestingly, for grade II (moderate) acute cholangitis, the prevalence was relatively constant for both COVID-19 (25%) and non-COVID-19 (32.1%) patients. For those with two bacterial types identified, the occurrence was almost equally distributed across all three types of Tokyo classification for both COVID-19 and non-COVID-19 categories, with a slightly higher incidence in severe cases (42.9% for COVID-19 and 31.8% for non-COVID-19). Although less common, cultures with three or more bacteria showed a 50% prevalence for both mild and severe Tokyo disease in the COVID-19 group versus a lower prevalence in the non-COVID-19 group.

We utilized binomial logistic regression to assess the link between various bacterial infections and COVID-19 status among patients with AC. The model showed that approximately 26.5% of the variance in COVID-19 status could be attributed to the differential bacterial profiles, as evidenced by an R-squared value of 0.26. This highlights the substantial role that bacterial infections play in the context of COVID-19 among this patient cohort. Among the various bacteria examined, *Pseudomonas* spp. was the only microorganism that showed a statistically significant relationship with the COVID-19 status of the patients. The results revealed an increasing odds ratio for *Pseudomonas* spp., suggesting that the presence of this bacteria is associated with a fourfold increase in the likelihood of being a COVID-19-positive case after adjusting for other bacterial infections. This finding highlights the importance of *Pseudomonas* spp. as a significant indicator of COVID-19 status in patients with cholangitis, highlighting its potential role in the pathophysiology of COVID-19-related complications in such populations.

Table 4 and Table 5 present a comprehensive analysis comparing the presence of various microorganisms in bile cultures of patients with COVID-19 and those without COVID-19. This comparison aims to identify significant differences in bacterial prevalence, which could inform treatment and management strategies for patients with AC during the pandemic.

Our findings indicate that *Pseudomonas* spp. exhibited a significant difference in occurrence rates between the two groups. Among patients with COVID-19, *Pseudomonas* spp. was identified in 16.7% of cases, compared to 5.7% in patients without COVID-19, with a *p*-value of 0.0281. This suggests a markedly higher prevalence of *Pseudomonas* spp. infections among patients suffering from COVID-19, highlighting a potential area of concern for managing secondary infections in these patients.

Other Gram-negative bacteria, such as *Escherichia coli* and *Klebsiella* spp., showed no statistically significant difference in prevalence between the two groups. *E. coli* was present in 36.7% of COVID-19 patients and 32.7% of non-COVID-19 patients, while *Klebsiella* spp. was found in 20.0% of COVID-19 patients compared to 14.7% in the control group.

Similarly, Gram-positive bacteria like *Enterococcus* spp. did not demonstrate significant differences in prevalence. *Enterococcus* spp. was observed in 23.3% of COVID-19 patients and 19.9% of non-COVID-19 patients.

## 4. Discussion

The present study offers a detailed analysis of AC’s etiology, clinical characteristics, and microbial profiles in COVID-19 infection. Our research highlights notable differences in the presentation and outcomes of AC patients with and without COVID-19, providing informative observations into the pandemic’s impact on the management and prognosis of biliary tract infections. The difference in mean age between COVID-19-positive and negative cohorts was statistically significant, with COVID-19 patients being notably older. This finding aligns with existing literature indicating that advanced age is a significant risk factor for severe COVID-19 outcomes [15,16,17]. Notably, gender distribution did not exhibit significant differences between the two groups, suggesting a lack of gender-specific predisposition to COVID-19 infection, consistent with previous findings [15,17].

Abdominal pain emerged as a significant symptom within the COVID-19-positive cohort, supporting emerging evidence that gastrointestinal manifestations, including abdominal pain, can be indicators of COVID-19 infection [16,18,19]. The presence of abdominal pain in COVID-19 patients warrants attention as a possible clinical marker for early detection and effective management strategies.

In contrast, no statistically significant difference was observed between the cohorts regarding fever. This finding contrasts with prior research emphasizing fever as a prevalent symptom in COVID-19 patients [16,17]. It underscores the heterogeneous nature of COVID-19’s clinical presentation and emphasizes the importance of comprehensive symptom evaluation in the diagnostic process [20].

In our study, patients who tested positive for COVID-19 experienced a significantly longer hospital stay, averaging 13.5 (6.6) days compared to 7.9 (5.4) days without COVID-19. This extended hospitalization period highlights the additional healthcare burden imposed by COVID-19. A recent study focusing on individuals with decompensated liver cirrhosis and COVID-19 revealed that the significant influence of COVID-19 on patients with LC, particularly concerning organ failure, associated infections, hospitalization, and mortality, was expected to some extent [11]. The severity of symptoms does not just determine extended hospitalization in COVID-19 cases; it is influenced by several factors, such as compromised functional status, referrals from other hospitals, specific admission criteria, chronic health conditions, and the emergence of complications during the hospital stay. Prolonged hospital stays are associated with COVID-19 and not exclusively with AC progression, as observed with other diseases [21].

Regarding comorbidities in the COVID-19 group, Type 2 diabetes, gender, and age were not significant predictors of hospitalization duration. In our analysis within the COVID-19 group, type 2 diabetes showed a weak positive correlation with hospitalization duration. However, another study revealed that COVID-19 patients with diabetes had a substantially longer hospital stay and a markedly higher incidence of ICU admissions compared to non-diabetic patients [22]. This discrepancy may be because the sample size in our research is fairly limited.

To accentuate the impact of COVID-19 on other pathologies, a recent study on acute pancreatitis during the pandemic reported that patients had a threefold increase in relative death risk compared to those before the pandemic [23]. These findings highlight the severe impact of the pandemic on patient outcomes and the increased risks associated with concurrent SARS-CoV-2 infection. A notable aspect of the study was the analysis of bile cultures, revealing distinct bacterial profiles in COVID-19 patients compared to their non-COVID counterparts. Sterile cultures were more prevalent among COVID-19 patients with mild Tokyo severity, while non-COVID patients with severe cases had a higher proportion of sterile cultures. This differential pattern suggests COVID-19 may influence the biliary microbial environment, potentially through immune modulation or direct viral effects on biliary tissue.

During the examination of various bacterial strains, *Pseudomonas* spp. emerged as significantly associated with COVID-19 status, showing a fourfold increased likelihood of presence in 16.7% of cases of COVID-19 patients (Odds Ratio = 4.2923, *p* = 0.018) compared to 5.7% in patients without COVID-19. This association highlights the need for increased vigilance and possibly specialized antimicrobial strategies in managing cholangitis in COVID-19 patients. The presence of *Pseudomonas* spp. as a significant indicator of COVID-19 status may reflect the opportunistic nature of this pathogen in immunocompromised or critically ill patients, a category in which COVID-19 patients often fall. However, the literature review did not yield data corresponding to these findings regarding AC.

Most studies examining bacterial coinfections in COVID-19 patients faced limitations due to insufficient sample sizes, hindering the ability to detect outcome differences between those with and without bacterial coinfection. However, a recent comprehensive analysis focusing on bacterial coinfections upon admission found that patients with bacterial coinfection had longer hospital stays and increased in-hospital mortality compared to those without. While not frequently detected upon admission, bacterial infections often emerged during the prolonged hospitalization of patients, with prevalent pathogens including *Pseudomonas aeruginosa*, *Klebsiella* spp., and *S. aureus* [24]. Numerous international reports have documented a slight uptick in the incidence of *Pseudomonas aeruginosa* bacteremia during the COVID-19 pandemic [25].

Other bacteria, including *E. coli* and *Klebsiella* spp., did not show significant differences between the groups, suggesting that while these pathogens are common in AC, their prevalence is not necessarily influenced by COVID-19. The lack of significant differences in these common pathogens may indicate that standard prophylactic and therapeutic measures remain effective for these bacteria, regardless of COVID-19 status.

In a comprehensive multicenter observational study conducted by Gomi et al. in 2017 focusing on patients with acute cholangitis, *E. coli* emerged as the predominant organism detected in bile cultures [26]. Consistent with these findings, review studies have also highlighted the prevalence of coliform organisms, including *Escherichia coli* (25–50%), *Klebsiella* spp. (15–20%), and *Enterobacter* species (5–10%) as commonly identified bacteria in AC cases [27,28,29].

Comparing our findings to the TG18 for AC reveals several deviations. While the guidelines delineate cholangitis severity based on clinical criteria such as systemic inflammation, cholestasis, and imaging findings, our study suggests that COVID-19 status may influence the microbial profile of acute cholangitis cases. This influence leads to varying patterns of bacterial growth and culture sterility across severity grades [4].

The findings of this study have several clinical implications. Firstly, the increased age and prolonged hospitalization of COVID-19-positive cholangitis patients necessitate special consideration for resource allocation and management strategies in healthcare settings. The higher incidence of abdominal pain and the distinct bacterial profiles, particularly the prevalence of *Pseudomonas* spp., emphasize the need for specific clinical protocols and potentially more aggressive management strategies for co-infected patients.

Moreover, the analysis of the microbial profiles suggests that routine bile culture and sensitivity testing should be emphasized in COVID-19-positive cholangitis patients to guide appropriate antimicrobial therapy. The distinct microbial landscape in these patients could impose more precise and effective treatment regimens, potentially improving outcomes and reducing the length of hospital stays. The global health landscape has been significantly impacted by the COVID-19 pandemic, primarily due to the prevalence of severe respiratory illness. Nonetheless, emerging data suggests that COVID-19 can also lead to secondary sclerosing cholangitis, commonly called post-COVID-19 cholangiopathy. This rare yet serious complication manifests as inflammation and damage to the bile ducts following a bout of COVID-19 infection [30].

While comprehensive, this study has several limitations that should be addressed in future research. The relatively small sample size of COVID-19-positive patients may limit the generalizability of the findings. Additionally, the study’s retrospective nature could introduce biases related to the accuracy of medical records and the diagnostic criteria used. Due to the retrospective design, it is challenging to ensure that all participants in the COVID-19 and non-COVID-19 groups had no previous infections before enrollment in the study. Our data is limited to what is recorded in our hospital’s database, and we acknowledge that this may not capture the entire infectious history of the patients. Many patients might have been diagnosed or treated for COVID-19 in other hospitals or by their primary care physicians, and these records are not readily available to us. Additionally, in this study, we did not have detailed information on the COVID-19 vaccination status of all participants. This lack of data on vaccination status is another limitation that could potentially affect the results and interpretation of our study.

## 5. Conclusions

Our study results show that COVID-19 affected the duration of hospitalization for patients with acute cholangitis. Furthermore, this study presents observations regarding the impact of COVID-19 on acute cholangitis, revealing differences in microbial profiles. The advanced age and prolonged hospitalization of these patients demand a more precise approach, while the increased incidence of abdominal pain and distinct bacterial profiles, particularly the prevalence of *Pseudomonas* spp., indicate the importance of developing specific clinical protocols. These observations suggest that a more intensive and individualized treatment strategy may be necessary to improve outcomes in this vulnerable patient population.

## Figures and Tables

**Table 1:** Distribution of acute cholangitis etiologies among COVID-19 positive and negative patients.

Condition	COVID (*n* = 30)	Without COVID (*n* = 211)	*p*-Value
**Benign**	13.0 (43.3%)	99.0 (46.9%)	
Choledocholithiasis	13.0 (43.3%)	92.0 (43.6%)	0.978 ^1^
Benign vaterian ampulloma	0.0 (0.0%)	2.0 (0.9%)	0.590 ^1^
Benign coledochal stenosis	0.0 (0.0%)	4.0 (1.9%)	0.447 ^1^
Liver abscess	0.0 (0.0%)	1.0 (0.5%)	0.706 ^1^
**Malignant**	17.0 (56.7%)	112.0 (53.1%)	
Pancreatic cancer	9.0 (30.0%)	61.0 (28.9%)	0.902 ^1^
Cholangiocarcinoma	6.0 (20.0%)	31.0 (14.7%)	0.450 ^1^
Malignant vaterian ampulloma	2.0 (6.7%)	13.0 (6.2%)	0.915 ^1^
Malignant extrinsic compression	0.0 (0.0%)	6.0 (2.8%)	0.350 ^1^
Gallbladder cancer	0.0 (0.0%)	1.0 (0.5%)	0.706 ^1^

*n*—number of patients; ^1^ Proportions are evaluated with a chi-square test.

**Table 2:** Clinical characteristics of the study population with biliary obstruction stratified by COVID-19 infection.

	COVID (*n* = 30)	Without COVID (*n* = 211)	*p* Value
Gender			0.539 ^1^
F	18.0 (60.0%)	114.0 (54.0%)	
M	12.0 (40.0%)	97.0 (46.0%)	
Age			**0.009 ^2^**
Mean (SD)	74.3 (10.6)	67.3 (14.1)	
Range	52.0–93.0	19.0–96.0	
Jaundice			0.918 ^1^
Yes	27.0 (90.0%)	192.0 (91%)	
No	3.0 (10.0%)	19.0 (9.0%)	
Abdominal pain			**0.025 ^1^**
Yes	27.0 (90.0%)	149.0 (70.6%)	
No	3.0 (10.0%)	62.0 (29.4%)	
Fever			0.264 ^1^
Yes	6.0 (20.0%)	63.0 (29.9%)	
No	24.0 (80.0%)	148.0 (70.1%)	
CRP (mg/L)			0.476 ^2^
Mean (SD)	119.65 (97.83)	105.70 (107.56)	
Range	11.0–322.8	2.14–545.9	
WBC (×10^3^/µL)			0.881 ^2^
Mean (SD)	11.73 (7.35)	11.52 (5.85)	
Range	3.61–40.0	2.81–41.9	
Total Bilirubin (mg/dL)			0.698 ^2^
Mean (SD)	10.77 (7.12)	10.22 (7.29)	
Range	1.6–30.2	0.5–36.3	
Platelets (×10^3^/µL)			0.959 ^2^
Mean (SD)	261.84 (120.52)	263.05 (109.51)	
Range	32.0–501.8	24.0–777.0	
INR			0.219 ^2^
Mean (SD)	1.52 (0.39)	1.42 (0.59)	
Range	0.96–2.77	0.91–5.05	
Cardiac pathology			0.058 ^1^
Yes	25 (83.3%)	135 (63.9%)	
No	5 (16.6%)	76 (36%)	
Type 2 Diabetes			0.727 ^1^
Yes	9 (30%)	53 (25.1%)	
No	21 (70%)	158 (74.9%)	
Chronic Kidney Disease			0.757 ^1^
Yes	0 (0%)	6 (2.8%)	
No	30 (100%)	205 (97.2%)	
Smoking status			
Ex-smoker	8 (30%)	64 (30.3%)	0.934 ^1^
Smoker	11 (36.7%)	63 (29.9%)	0.635 ^1^
Non-smoker	10 (33.3%)	84 (39.8%)	0.723 ^1^
Previous stent			0.362 ^1^
Yes	7.0 (23.3%)	35.0 (16.6%)	
No	23.0 (76.7%)	176.0 (83.4%)	
Hospitalization days			**<0.001 ^2^**
Mean (SD)	13.5 (6.6)	7.9 (5.4)	
Range	4.0–26.0	1.0–35.0	
Weekend admission			0.550 ^1^
Yes	8.0 (26.7%)	46.0 (21.8%)	
No	22.0 (73.3%)	165.0 (78.2%)	
Tokyo severity score			0.103 ^1^
Grade I	10.0 (33.3%)	85.0 (40.3%)	
Grade II	6.0 (20.0%)	67.0 (31.8%)	
Grade III	14.0 (46.7%)	59.0 (28.0%)	

*n*—number of patients; ^1^ Proportions are evaluated with a chi-square test; ^2^ Linear Model ANOVA; CRP—C-reactive protein; WBC-white blood cells; SD—Standard Deviation, INR—international normalized ratio.

**Table 3:** Bacterial presence in bile specimens: Insights from the Tokyo Guidelines Severity Grades and COVID-19 Status.

		Sterile			1 Bacterium			2 Bacteria			>3 Bacteria	
	COVID	Non-COVID	*p*	COVID	Non-COVID	*p*	COVID	Non-COVID	*p*	COVID	Non-COVID	*p*
Tokyo Grade I	3 (60%)	30 (40%)		4 (25%)	35 (41.7%)		2 (28.6%)	18 (40.90%)		1 (50%)	2 (25%)	
Tokyo Grade II	0	25 (33.3%)	0.298 ^1^	4 (25%)	27 (32.1%)	0.156 ^1^	2 (28.6%)	12 (27.30%)	0.795 ^1^	0	3 (37.5%)	0.429 ^1^
Tokyo Grade III	2 (40%)	20 (26.7%)		8 (50%)	22 (26.2%)		3 (42.9%)	14 (31.80%)		1 (50%)	3 (37.5%)	

^1^ Proportions are evaluated with a chi-square test.

**Table 4:** Binominal Logistic Regression Analysis of Bacterial Profiles at patients with acute cholangitis based on COVID-19 status.

						95% Confidence Interval	
Predictor	Estimate	SE	Z	*p*	Odds Ratio	Lower	Upper
Intercept	−2.494	0.339	7.356	**<** **0** **.001**	0.0826	0.0425	0.160
Other bile germs:							
Yes—No	0.999	0.627	1.593	0.111	2.7147	0.7945	9.275
*Acinetobacter* spp.							
Yes—No	0.531	1.181	0.450	0.653	1.7014	0.1682	17.205
*Citrobacter* spp.							
Yes—No	0.816	0.828	0.985	0.324	2.2608	0.4462	11.454
*Pseudomonas* spp.							
Yes—No	1.457	0.615	2.369	**0.018**	4.2923	1.2857	14.329
*Enterococcus* spp.							
Yes—No	0.320	0.488	0.655	0.512	1.3771	0.5289	3.586
*Klebsiella* spp.							
Yes—No	0.261	0.514	0.509	0.611	1.2988	0.4742	3.558
*Escherichia coli*:							
Yes—No	0.337	0.431	0.782	0.434	1.4008	0.6021	3.259

Note: Estimates represent the log odds of “Group = COVID” vs. “Group = Without COVID” (dependent variable). The predictors listed are considered independent variables. The model was not adjusted for gender and age. Each estimate is accompanied by its standard error (SE) and Z-score (Z), providing insight into each predictor’s statistical significance and stability within the model. ‘Yes vs. No’ denotes the presence vs. absence of specific bacterial species.

**Table 5:** Impact of COVID-19 on the prevalence of microbial germs in bile cultures.

	COVID (*n* = 30)	Without COVID (*n* = 211)	*p*-Value
Other bile germs			0.237 ^1^
No	26.0 (86.7%)	196.0 (92.9%)	
Yes	4.0 (13.3%)	15.0 (7.1%)	
*Acinetobacter* spp.			0.605 ^1^
No	29.0 (96.7%)	207.0 (98.1%)	
Yes	1.0 (3.3%)	4.0 (1.9%)	
*Citrobacter* spp.			0.555 ^1^
No	28.0 (93.3%)	202.0 (95.7%)	
Yes	2.0 (6.7%)	9.0 (4.3%)	
*Pseudomonas* spp.			0.028 ^1^
No	25.0 (83.3%)	199.0 (94.3%)	
Yes	5.0 (16.7%)	12.0 (5.7%)	
*Enterobacter* spp.			0.200 ^1^
No	30.0 (100.0%)	200.0 (94.8%)	
Yes	0.0 (0.0%)	11.0 (5.2%)	
*Enterococcus* spp.			0.662 ^1^
No	23.0 (76.7%)	169.0 (80.1%)	
Yes	7.0 (23.3%)	42.0 (19.9%)	
*Klebsiella* spp.			0.450 ^1^
No	24.0 (80.0%)	180.0 (85.3%)	
Yes	6.0 (20.0%)	31.0 (14.7%)	
*Streptococcus* spp.			0.350 ^1^
No	30.0 (100.0%)	205.0 (97.2%)	
Yes	0.0 (0.0%)	6.0 (2.8%)	
*Escherichia coli*			0.666 ^1^
No	19.0 (63.3%)	142.0 (67.3%)	
Yes	11.0 (36.7%)	69.0 (32.7%)	

*n*—number of patients; ^1^ Proportions are evaluated with a chi-square test.

## Data Availability

Data are available upon request.
